# A nomogram predicts early neurological deterioration after mechanical thrombectomy in patients with ischemic stroke

**DOI:** 10.3389/fneur.2023.1255476

**Published:** 2023-09-20

**Authors:** Kongyuan Wu, Zhengzhou Yuan, Wenhuo Chen, Tingyu Yi, Xiwen Chen, Mengmeng Ma, Jian Guo, Muke Zhou, Ning Chen, Li He

**Affiliations:** ^1^Department of Neurology, West China Hospital, Sichuan University, Chengdu, China; ^2^Department of Neurology, The Affiliated Hospital of Southwest Medical University, Luzhou, China; ^3^Department of Neurology, Zhangzhou Municipal Hospital of Fujian Province and Zhangzhou Affiliated Hospital of Fujian Medical University, Zhangzhou, China

**Keywords:** acute ischemic stroke, mechanical thrombectomy, prediction scale, early neurological deterioration, nomogram

## Abstract

**Introduction:**

Early neurological deterioration (END) is common in acute ischemic stroke and is directly associated with poor outcome after stroke. Our aim is to develop and validate a nomogram to predict the risk of END after mechanical thrombectomy (MT) in acute ischemic stroke patients with anterior circulation large-vessel occlusion.

**Methods:**

We conducted a real-world, multi-center study in patients with stroke treated with mechanical thrombectomy. END was defined as a worsening by 2 or more NIHSS points within 72-hour after stroke onset compared to admission. Multivariable logistic regression was used to determine the independent predictors of END, and the discrimination of the scale was assessed using the C-index. Calibration curves were constructed to evaluate the calibration of the nomogram, and decision curves were used to describe the benefits of using the nomogram.

**Results:**

A total of 1007 patients were included in our study. Multivariate logistic regression analysis found age, admission systolic blood pressure, initial NIHSS scores, history of hyperlipemia, and location of occlusion were independent predictors of END. We developed a nomogram that included these 6 factors, and it revealed a prognostic accuracy with a C-index of 0.678 in the derivation group and 0.650 in the validation group. The calibration curves showed that the nomogram provided a good fit to the data, and the decision curves demonstrated a large net benefit.

**Discussion:**

Our study established and validated a nomogram to stratify the risk of END before mechanical embolectomy and identify high-risk patients, who should be more cautious when making clinical decisions.

## 1. Introduction

Mechanical thrombectomy (MT) has been proven to be an effective treatment for acute ischemic stroke (AIS) with anterior circulation large-vessel occlusion and was established as the new standard of care in recent years (1–3). Nonetheless, there is still a significant proportion of patients who fail to achieve favorable outcomes, and 28.6–67.8% of patients have poor functional outcomes after MT therapy (4). Previous studies have found several factors that could potentially affect the prognosis after mechanical thrombectomy (5), including early neurological deterioration (END) (6, 7).

END is common in acute ischemic stroke, occurs in 10–40% of patients (8), and is directly associated with poor long-term outcomes after stroke (5, 6, 9). Adjusted for factors such as age, sex, initial stroke severity, and admission time, END increases the risk of poor outcomes at 3 months by approximately 8–34 times and increases mortality by 5-fold (8, 10).

Therefore, early identification of high-risk individuals with possible END will be helpful for clinicians to judge the prognosis after MT and to select the most appropriate candidates for MT therapy. Early intervention of the risk factors of END could possibly reduce the risk of adverse prognosis and improve functional outcomes that make patients benefit more from MT.

Several studies on END among patients treated with MT have found some independent predictors of END, such as age, admission systolic blood pressure, successful recanalization, and occlusion site (6, 7, 11, 12). However, there is not yet a suitable tool for clinicians to predict the risk of END before surgery.

Our study aimed to develop and validate a nomogram based on multi-center real-world data from Chinese patients to predict the risk of END after thrombectomy (direct or bridging thrombectomy) in AIS patients with anterior circulation large-vessel occlusion.

## 2. Methods

### 2.1. Study design and participants

We conducted a real-world, multi-center, retrospective case–control study in acute ischemic stroke patients treated with mechanical thrombectomy in West China Hospital, the Affiliated Hospital of Southwest Medical University, Mianyang Central Hospital, and Zigong Third People’s Hospital between January 2015 and April 2021.

The inclusion criteria were as follows: (1) age ≥ 18 years, (2) acute ischemic stroke (AIS), defined by the World Health Organization criteria and the Chinese guidelines, (3) treatment with mechanical thrombectomy, and (4) occlusion of large vessels, including the internal carotid artery (ICA), first/s/third segment of the middle cerebral artery (M1/M2/M3), or the anterior cerebral artery (A), confirmed by computed tomographic angiography (CTA), magnetic resonance angiography (MRA), or digital subtraction angiography (DSA).

The exclusion criteria were as follows: (1) intracranial hemorrhage confirmed by computed tomography (CT) or magnetic resonance imaging (MRI) before MT, (2) involvement of both the anterior and posterior circulation, and (3) incomplete clinical data.

### 2.2. Data collection

Demographic information and clinical data of each patient were collected, including age, sex, smoking status, disease history (i.e., previous stroke, hypertension, hyperlipemia, diabetes mellitus (DM), and atrial affiliation (AF)), laboratory characteristics (i.e., systolic blood pressure, diastolic blood pressure, and admission blood glucose), stroke subtype classified by TOAST, NIHSS score, ASPECT score, and procedural characteristics (i.e., anesthesia, location of occlusion, collateral flow (ASTIN/SIR), embolectomy devices, time from onset to groin puncture, time from groin puncture to successful recanalization, modified thrombolysis in cerebral infarction (mTICI) scale, and number of passes).

### 2.3. Outcomes and follow-Up

The primary outcome was early neurological deterioration (END), which was defined as a worsening by two or more NIHSS points within 72 h after stroke onset compared to admission (13). The evaluation of the NIHSS score was conducted by certificated investigators who were not aware of the study design.

Patients were evaluated carefully since admission and were followed up by face-to-face assessments in the outpatient department of each hospital or by telephone interviews.

### 2.4. Statistical analysis

Patients were randomly assigned to a derivation group (60%) and a validation group (40%). Continuous variables were described as median and interquartile range (IQR) values, and categorical variables were described as counts and percentages. Student’s *t*-test was used for normally distributed continuous variables, and the Mann–Whitney U test was used for those that did not conform to a normal distribution. Categorical variables were assessed by χ2 or Fisher’s exact test.

Factors with a *p*-value of <0.1 in the univariate analysis were further entered into the multivariable logistic regression analysis, and factors with a *p*-value of <0.05 in the multivariable logistic regression analysis were considered to be independent risk factors for END. Odds ratios and 95% confidence intervals (CIs) were calculated. The nomogram was developed based on the independent risk factors.

The discrimination of the model was assessed by the C statistics. Calibration curves were constructed to evaluate the calibration of the nomogram. The decision curve was used to describe the net benefits and medical interventions from using the nomogram.

Statistical analysis was performed using SPSS version 23.0 and FreeStatistics version 1.5.1.

## 3. Results

**Figure 1:**
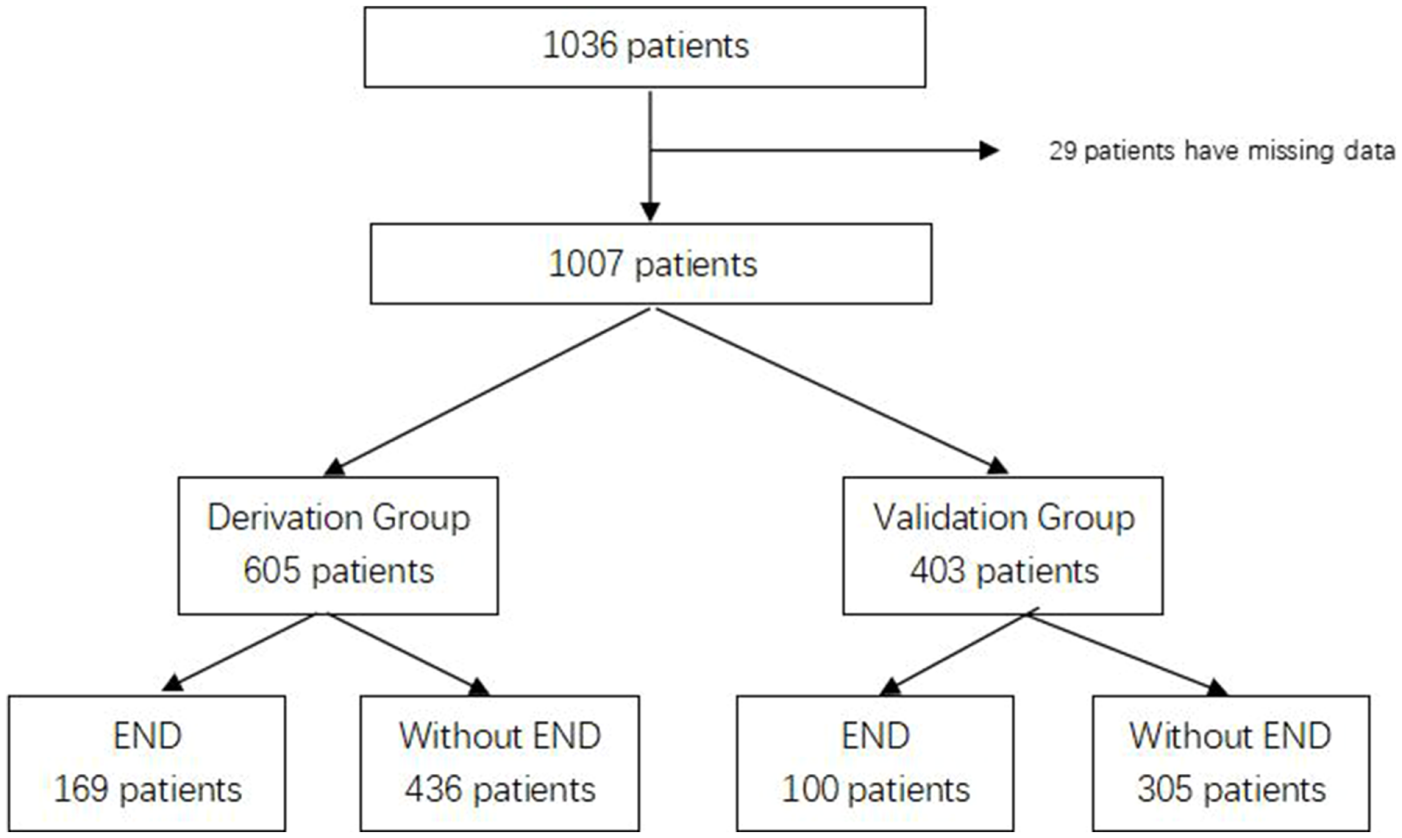
Flow chart.

Between January 2015 and April 2021, we collected information on 1,036 patients who met the inclusion criteria and excluded 29 patients with missing data. A total of 1,007 patients were enrolled in our study and randomly assigned to the derivation group (*n* = 605) and the validation group (*n* = 403). The flow chart is shown in Figure 1. END was found related to poor functional outcomes in 90 days with an OR of 7.477 (95% CI 4.865–11.490, *p* < 0.001). The baseline characteristics of both groups are shown in Table 1. There were 110 patients (18.2%) in the derivation group and 97 patients (24.1%) in the validation group who had a history of diabetes, and other characteristics showed no significant differences.

**Table 1:** Baseline characteristics of patients in the derivation group and validation group.

Variables	Total (*n* = 1,007)	Derivation Group (*n* = 605)	Validation Group (*n* = 402)	*p* value
Sex, male, *n* (%)	523 (51.9)	315 (52.1)	208 (51.7)	0.971
Age, *n* (%)				0.402
< 60	234 (23.2)	145 (24)	89 (22.1)	
60–80	637 (63.3)	373 (61.7)	264 (65.7)	
≥ 80	136 (13.5)	87 (14.4)	49 (12.2)	
Smoke, *n* (%)	251 (24.9)	155 (25.6)	96 (23.9)	0.582
Hypertension, *n* (%)	626 (62.2)	369 (61)	257 (63.9)	0.381
Diabetes, *n* (%)	207 (20.6)	110 (18.2)	97 (24.1)	0.027
Hyperlipemia, *n* (%)	187 (18.6)	104 (17.2)	83 (20.6)	0.194
Atrial fibrillation, *n* (%)	455 (45.2)	274 (45.3)	181 (45)	0.986
Systolic blood pressure, mmHg, *n* (%)				0.539
<140	534 (53.0)	320 (52.9)	214 (53.2)	
140–160	236 (23.4)	150 (24.8)	86 (21.4)	
160–180	168 (16.7)	96 (15.9)	72 (17.9)	
≥180	69 (6.9)	39 (6.4)	30 (7.5)	
Admission blood glucose, mmol/L, *n* (%)				0.536
<11.1	915 (90.9)	553 (91.4)	362 (90)	
≥11.1	92 (9.1)	52 (8.6)	40 (10)	
ASPECTS, *n* (%)				0.929
<6	73 (7.2)	43 (7.1)	30 (7.5)	
≥6	934 (92.8)	562 (92.9)	372 (92.5)	
NIHSS, Median (IQR)	16.0 (12.0, 19.0)	15.0 (12.0, 19.0)	16.0 (12.0, 19.8)	0.975
Thrombolysis, *n* (%)	193 (19.2)	118 (19.5)	75 (18.7)	0.8
Time from onset-to-puncture, Median (IQR)	290.0 (222.5, 399.0)	297.0 (223.0, 409.0)	275.5 (220.0, 385.2)	0.086
Anesthesia, *n* (%)				0.569
Local anesthesia	584 (58.0)	346 (57.2)	238 (59.2)	
General anesthesia	423 (42.0)	259 (42.8)	164 (40.8)	
ASITNSIR < 2, *n* (%)	564 (56.0)	332 (54.9)	232 (57.7)	0.411
TOAST, *n* (%)				0.434
Cardioembolism	637 (63.3)	390 (64.5)	247 (61.4)	
Large-artery atherosclerosis	278 (27.6)	165 (27.3)	113 (28.1)	
other and undermined	92 (9.1)	50 (8.3)	42 (10.4)	
Location of occlusion, *n* (%)				0.805
ICA	245 (24.3)	147 (24.3)	98 (24.4)	
M1	516 (51.2)	303 (50.1)	213 (53)	
M2	184 (18.3)	114 (18.8)	70 (17.4)	
M3	35 (3.5)	23 (3.8)	12 (3)	
A	27 (2.7)	18 (3)	9 (2.2)	
Number of passes, mean ± SD	2.8 ± 1.6	2.8 ± 1.6	2.8 ± 1.6	0.897
Reperfusion, *n* (%)	848 (84.2)	509 (84.1)	339 (84.3)	1
END, *n* (%)	269 (26.7)	169 (27.9)	100 (24.9)	0.317

ICA, internal carotid artery; M1, first segment of middle cerebral artery; M2, second segment of middle cerebral artery; M3, third segment of middle cerebral artery; A, anterior cerebral artery.

### 3.1. Derivation

**Figure 2:**
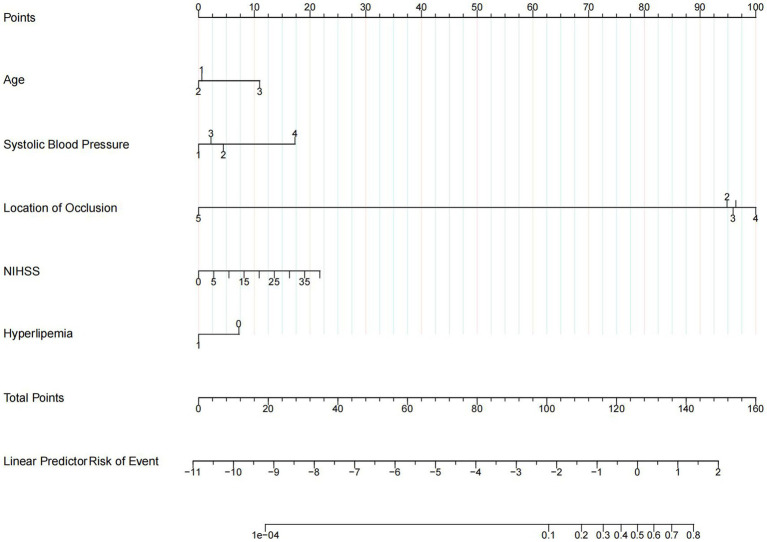
Nomogram. Nomogram predicts early neurological deterioration after endovascular thrombectomy in patients with ischemic stroke. For age, 1 means age < 60 years, 2 means age ≥ 60 and < 80 years, 3 means age ≥ 80 years. For systolic blood pressure (SBP), 1 means SBP < 140, 2 means SBP ≥ 140 and < 160, 3 means SBP ≥ 160 and < 180, and 4 means SBP ≥ 180. For the location of occlusion, 1 means internal carotid artery, 2 means M1 of the middle cerebral artery, 3 means M2 of the middle cerebral artery, 4 means M3 of the middle cerebral artery, and 5 means arteria cerebri anterior. For hyperlipemia, 1 means patients with a history of hyperlipemia and 0 means patients without a history of hyperlipemia.

The results of univariate logistic regression analysis and multivariate logistic regression analysis are shown in Table 2; factors that were available pre-puncture with *p* < 0.1 in the univariate analysis were further entered into the multivariable logistic regression analysis. Age (age 60–80: adj. OR = 0.97, 95% CI = 0.67–1.41, age ≥ 80: adj. OR = 2.11, 95% CI = 1.30–3.42), admission systolic blood pressure (SBP 140–160: adj. OR = 1.34, 95% CI = 0.93–1.93, SBP 160–180: adj. OR = 1.09, 95% CI = 0.71–1.65, SBP ≥180: adj. OR = 2.98, 95% CI = 1.71–5.19), initial NIHSS score (adj. OR = 1.03, 95% CI = 1.01–1.05), history of hyperlipemia (adj. OR = 0.62, 95% CI = 0.41–0.93), and location of occlusion site (M1: adj. OR = 0.64, 95% CI = 0.45–0.91, M2: adj. OR = 0.74, 95% CI = 0.48–1.15, M3: adj. OR = 1.19, 95% CI = 0.53–2.67, A: adj. OR = 0.09, 95% CI = 0.01–0.68) were independent risk factors for END. A nomogram for estimating the risk of END consisted of the abovementioned 5 variables (Figure 2).

**Table 2:** Univariate analysis and multivariate logistic regression analysis of patients with END and without END in the derivation group.

Variable	OR	95% CI		*p* value	Adjusted OR	Adjusted OR 95% CI		Adjusted *p*-value
		Lower	Upper			Lower	Upper	
Sex, male	0.76	0.57	1	0.051	0.85	0.63	1.15	0.289
Age								
Age < 60	1 (Ref)				1 (Ref)			
Age 60–80	1.08	0.76	1.54	0.666	0.97	0.67	1.41	0.869
Age ≥ 80	2.55	1.62	4.03	<0.001	2.11	1.30	3.42	0.002
Systolic blood pressure								
SBP < 140	1 (Ref)				1 (Ref)			
SBP 140–160	1.35	0.96	1.91	0.086	1.34	0.93	1.93	0.113
SBP 160–180	1.13	0.75	1.68	0.562	1.09	0.71	1.65	0.696
SBP ≥ 180	2.83	1.69	4.73	<0.001	2.98	1.71	5.19	<0.001
Admission blood glucose ≥ 11.1	1.29	0.81	2.06	0.275				
NIHSS	1.03	1.01	1.05	0.008	1.03	1.00	1.05	0.023
ASPECTS ≥ 6	0.52	0.32	0.86	0.01	0.70	0.41	1.18	0.178
Smoke	0.92	0.66	1.27	0.616				
Hypertension	1.52	1.13	2.04	0.006	1.32	0.96	1.80	0.087
Diabetes	1.38	0.99	1.92	0.06	1.40	0.98	2.00	0.068
Hyperlipemia	0.58	0.39	0.86	0.007	0.62	0.41	0.93	0.023
Atrial fibrillation	1.32	0.99	1.74	0.054	1.28	0.95	1.73	0.106
Thrombolysis	1.01	0.71	1.45	0.936				
Time from onset-to-puncture	1	1	1	0.63				
General anesthesia	1.13	0.85	1.5	0.386				
ASITNSIR < 2	1.91	1.42	2.55	<0.001				
TOAST								
Cardioembolism	1 (Ref)							
Large-artery atherosclerosis	0.47	0.33	0.66	<0.001				
Other and undermined	0.41	0.23	0.74	0.003				
Location of occlusion								
ICA	1 (Ref)				1 (Ref)			
M1	0.65	0.46	0.9	0.011	0.64	0.45	0.91	0.012
M2	0.78	0.51	1.18	0.236	0.74	0.48	1.15	0.179
M3	0.93	0.43	1.99	0.847	1.19	0.53	2.67	0.674
A	0.08	0.01	0.58	0.013	0.09	0.01	0.68	0.020
Number of passes	1.28	1.17	1.39	<0.001				
Reperfusion	0.46	0.32	0.65	<0.001				

### 3.2. Validation

**Figure 3:**
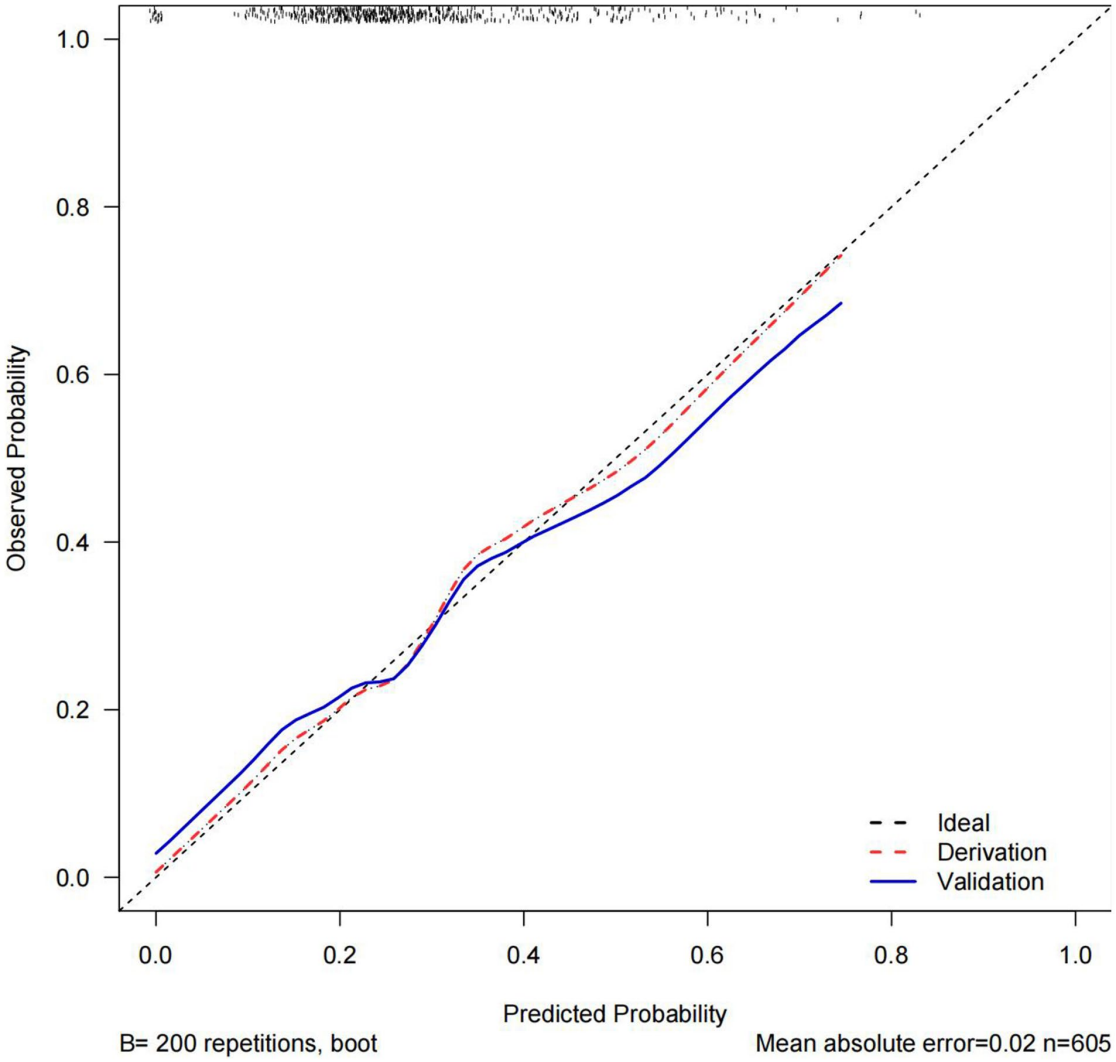
Calibrate curves. Calibration curves for the derivation and validation groups.

**Figure 4:**
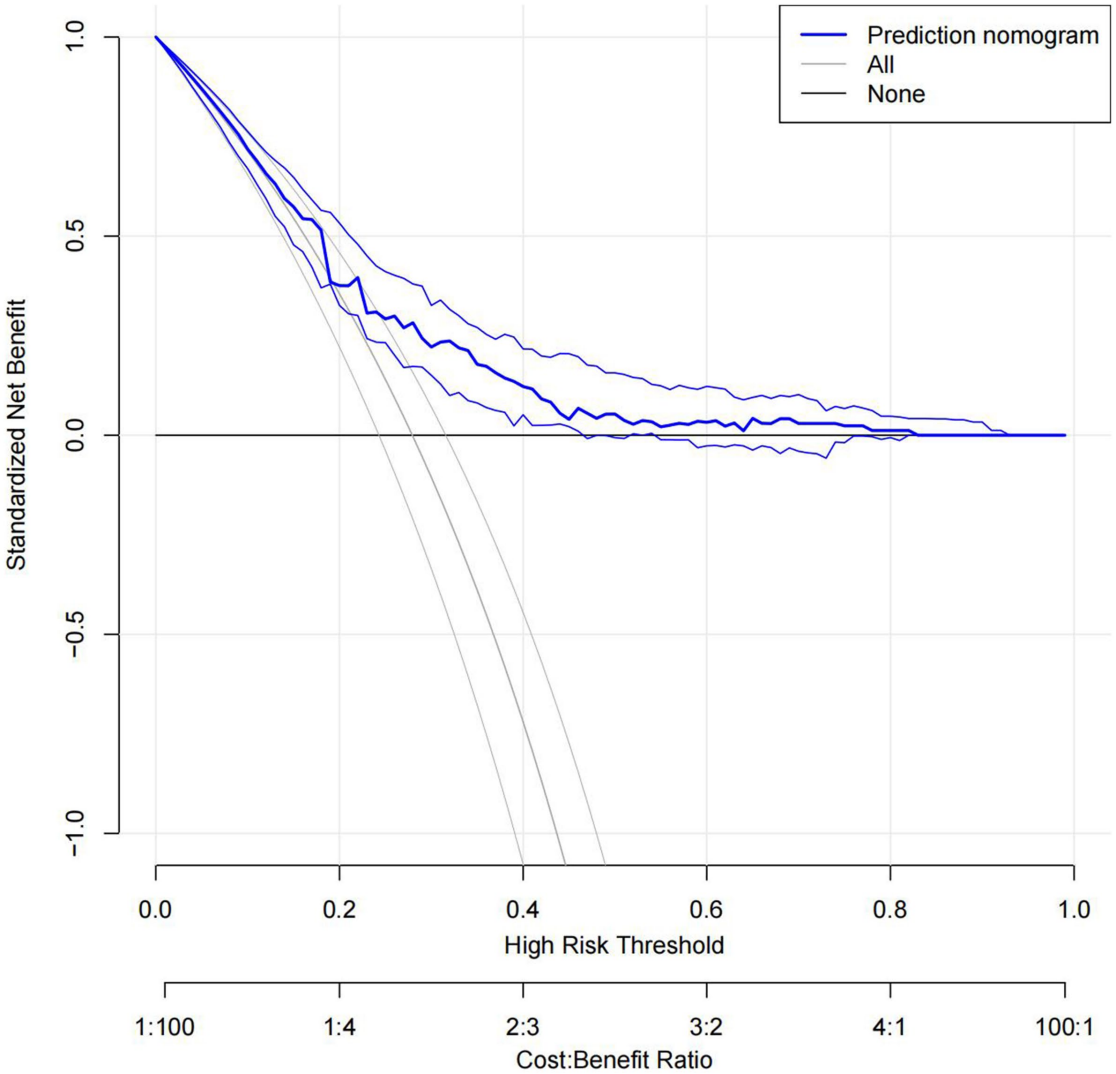
Decision curves. Decision curves of the derivation group.

The C statistics of this model is 0.678 for the derivation group and 0.650 for the validation group. The calibration curves of the nomogram are shown in Figure 3; all are almost diagonal, indicating that the nomogram provides a good fit to the data. Figure 4 shows the decision curves of the nomogram. This finding demonstrates that this nomogram provides a larger net benefit when the probability is between 0 and 0.8.

## 4. Discussion

In this study, we developed and validated a nomogram to predict END after MT in patients with anterior circulation acute ischemic stroke treated with mechanical thrombectomy. The nomogram consists of 5 preoperative variables—age, admission systolic blood pressure, initial NIHSS score, history of hyperlipemia, and location of occlusion site. The discrimination and calibration of the nomogram were good in both the derivation and validation groups. The decision curves show the good clinical usefulness of the nomogram and demonstrate a large net benefit when the probability was between 0 and 0.8.

Previous studies have found that END is common in acute ischemic stroke (8) and is directly associated with poor outcomes and higher mortality (6, 7). Possible mechanisms were symptomatic intracerebral hemorrhage, ischemic progression, reembolization, malignant edema, and partially unexplained (12, 14). Although numerous risk factors for END have been found (6, 11), several models have been established to predict long-term prognosis after MT (15–17) and to predict END after IVT (12). However, there is not yet a suitable tool for clinicians to predict the risk of END before surgery.

Moreover, there is not yet a standardized definition of early neurologic deterioration (END), and previous studies using different definitions of END may achieve contrasting conclusions (14). It was shown to be a highly sensitive predictor of poor functional outcome of a ≥ 2-point change in NIHSS, while ≥4 points were highly specific (13). END is concentrated during the acute period, especially within 72 h after thrombectomy (6). In our studies, we aimed to identify high-risk patients with END earlier; therefore, we tended to choose the highly sensitive definition of a ≥ 2-point change in 72 h.

It was found that although elderly patients had the same benefit of endovascular treatment as younger patients, functional outcomes worsened with increasing age (18). Additionally, Girot et al. found a high risk of END in elderly patients (11), which is consistent with the results of our study. A possible explanation is that preexisting disability (19) and comorbidities (11, 20) are more common in elderly patients. Decreased functional reserve and diminished tolerance to larger core infarcts (18) also contribute to poor outcomes.

We found an association between high-admission SBP and END. This was consistent with previous studies that found that patients with higher levels of SBP were at increased risk of 90-day poor outcomes (21, 22) and early neurological deterioration (23). Additionally, an increased occurrence of sICH and lower rates of successful reperfusion were found in patients with higher SBP, which is a possible explanation for the association between higher SBP and poor outcomes (21).

Consistent with previous results, ICA occlusion was an independent predictor of END (12, 24). One possible explanation is that the proximal occlusion site predicts stroke severity and the risk of further extension of the penumbra and ischemia progression (6). Additionally, it has been reported that ICA occlusion is a risk factor for reocclusion after MT (25), which is one of the potential mechanisms of END (26).

We found that the initial NIHSS score was an independent risk predictor of END, which is consistent with previous studies (27–29), but there were also reports that low initial NIHSS scores were associated with a higher risk of END. One possible reason was different definitions of END (14), and another potential explanation is that the NIHSS reflects neurological deficits but not perfusion. Patients with lower NIHSS scores may have larger ischemic penumbras leading to infarction progression (11, 30).

We demonstrated that patients without a history of hyperlipemia had a higher risk of END. Although hyperlipemia was a risk factor for ischemic stroke (31), it has been reported that lower serum triglyceride and cholesterol levels were related to poor outcomes after acute ischemic stroke, larger infarct volume, and increased stroke severity (26, 32). One of the potential mechanisms of this association is that lower serum TG may reflect poor individual nutritional status, which is a risk factor for poor outcomes after AIS (26). Another possible explanation is the antioxidant protection of cholesterol through increasing gamma glutamyltransferase (33).

We also found that cardioembolism predicts a higher risk of END. It was shown in one study of END with isolated ICA occlusion that END–non-atheromatous patients were at much higher END risk than atheromatous patients (30). In our studies, we performed a preliminary assessment of TOAST by radiology (e.g., CTA) and medical history (e.g., history of atrial fibrillation and history of hypertension). However, the exact classification of TOAST is usually uncertain in the emergency evaluation and will be assessed in detail during hospitalization, therefore we did not include it in the prediction model. We also performed a model with TOAST classification, which showed values of 0.709 and 0.681 of C statistics in the derivation and validation group, which may show the importance of TOAST in predicting END.

Our study also has limitations. Our aim was to assess END early before surgery, and we only included preoperative factors. Factors such as successful recanalization (6) and times to pass (11), which were also found to be independently associated with END after MT in this study were excluded because they were not available before puncture. However, there was a strong association between these two factors and END, with an unadjusted OR of 0.46 (95% CI 0.32–0.65, *p* < 0.001) for successful recanalization and 1.28 (95% CI 1.17–1.39, *p* < 0.001) for the number of passes. For a similar reason, TOAST was also excluded. This may explain the unfavorable predictive effectiveness of this model. This finding indicated that for patients with a predicted high risk of END, it should be more cautious to perform thrombectomy repeatedly during the operation. We performed further analyses that found common risk factors in both subgroups with successful reperfusion and without reperfusion, which is consistent with previous results of our analysis. The results are shown in the Supplementary Table S1. We also found different factors in the two subgroups which may explain the possible mechanisms of END in each group. In the group without successful reperfusion, thrombolysis before mechanical thrombectomy was found related to a lower risk of END, for the reason that the possible mechanism is infarction extension. While in a group with successful reperfusion, END may be associated with malignant edema and symptomatic intracranial hemorrhage caused by ischemia/reperfusion injury. However, it is not the main subject of our study, so we did not state much about it. Future studies should be performed to analyze the impact of the above results and find more preoperative risk factors and then improve the prediction model. Second, this prediction model was validated internally only. Future studies are needed to perform external validation to improve accuracy and sensitivity. Third, this was a retrospective study, and we excluded patients with missing data, which may have introduced selection bias and confounding bias. Finally, the participants were from China, and the results could not be easily extrapolated to other ethnic groups, and it needs future studies to validate the model in other populations.

In conclusion, our study developed and validated a simple and practical preoperative END prediction nomogram that can stratify END risk before mechanical thrombectomy which helps to screen high-risk patients with END. It provides a reference for preoperative clinician decision-making, doctor–patient communication, and optimization of postoperative management but should be interpreted with caution.

## Data availability statement

The raw data supporting the conclusions of this article will be made available by the authors, without undue reservation.

## Ethics statement

The study involving human participants were reviewed and approved by the ethics committee of West China Hospital, Sichuan University [No.2019 (319)]. Written informed consent from the patients/participants or patients/participants’ legal guardian/next of kin was not required to participate in this study since all data were retrospectively collected and individual information were not disclosed.

## Author contributions

KW: Data curation, Formal analysis, Writing – original draft. ZY: Data curation, Formal analysis, Writing – original draft, Investigation, Methodology. WC: Data curation, Investigation, Writing – review & editing. TY: Data curation, Investigation, Writing – review & editing. XC: Data curation, Investigation, Writing – review & editing. MM: Data curation, Investigation, Writing – review & editing. JG: Data curation, Investigation, Writing – review & editing. MZ: Data curation, Investigation, Writing – review & editing. NC: Conceptualization, Data curation, Methodology, Project administration, Supervision, Validation, Writing – review & editing. LH: Data curation, Funding acquisition, Investigation, Methodology, Resources, Supervision, Validation, Writing – review & editing.

## Funding

The study is supported by the Sichuan Science and Technology Program (2019YFH0196), the 1·3·5 project for disciplines of excellence–Clinical Research Incubation Project, West China Hospital, Sichuan University (2018HXFH022), and the National Natural Science Foundation of China (81500959).

## Conflict of interest

The authors declare that the research was conducted in the absence of any commercial or financial relationships that could be construed as a potential conflict of interest.

## Publisher’s note

All claims expressed in this article are solely those of the authors and do not necessarily represent those of their affiliated organizations, or those of the publisher, the editors and the reviewers. Any product that may be evaluated in this article, or claim that may be made by its manufacturer, is not guaranteed or endorsed by the publisher.
