# Supplementary material for: A nomogram predicts early neurological deterioration after mechanical thrombectomy in patients with ischemic stroke
Source: Front Neurol. 2023 Sep 20;14:1255476. doi: 10.3389/fneur.2023.1255476 (PMC10548384; doi:10.3389/fneur.2023.1255476)
Supplement: Supplementary file 1 [file Table_1.docx]

**Supplementary table:** Subgroups analysis of group with successful reperfusion and without successful reperfusion

| **Variable** | **Successful Reperfusion** | | | | **Without Reperfusion** | | | |
| --- | --- | --- | --- | --- | --- | --- | --- | --- |
|  | **OR** | **95% CI** | | **P Value** | **OR** | **95% CI** | | **P Value** |
|  |  | **Lower** | **Upper** |  |  | **Lower** | **Upper** |  |
| **Gender, male** | 0.741 | 0.541 | 1.017 | 0.063 | 0.837 | 0.445 | 1.577 | 0.583 |
| **Age** |  |  |  |  |  |  |  |  |
| **Age <60** | 1(Ref) |  |  |  | 1(Ref) |  |  |  |
| **Age 60-80** | 1.279 | 0.846 | 1.935 | 0.243 | 0.731 | 0.345 | 1.548 | 0.413 |
| **Age ≥80** | 2.782 | 1.636 | 4.732 | <0.001 | 1.968 | 0.757 | 5.116 | 0.165 |
| **Systolic Blood Pressure** | 1.007 | 1.001 | 1.014 | 0.025 | 1.029 | 1.014 | 1.045 | <0.001 |
| **Admission Blood Glucose≥**11.1 | 1.67 | 1.03 | 2.71 | 0.038 | 0.194 | 0.023 | 1.618 | 0.13 |
| **NIHSS** | 1.047 | 1.02 | 1.075 | 0.001 | 0.999 | 0.942 | 1.06 | 0.969 |
| **ASPECTS ≥**6 | 0.876 | 0.799 | 0.96 | 0.005 | 0.707 | 0.567 | 0.88 | 0.002 |
| **Smoke** | 0.944 | 0.651 | 1.371 | 0.764 | 0.686 | 0.341 | 1.38 | 0.291 |
| **Hypertension** | 1.622 | 1.153 | 2.281 | 0.006 | 1.38 | 0.722 | 2.636 | 0.33 |
| **Diabetes** | 1.633 | 1.138 | 2.344 | 0.008 | 0.739 | 0.294 | 1.861 | 0.521 |
| **Hyperlipemia** | 0.688 | 0.446 | 1.061 | 0.091 | 0.257 | 0.092 | 0.718 | 0.01 |
| **Atrial Fibrillation** | 1.102 | 0.803 | 1.513 | 0.547 | 2.218 | 1.107 | 4.093 | 0.024 |
| **Thrombolysis** | 1.243 | 0.85 | 1.817 | 0.262 | 0.406 | 0.141 | 1.172 | 0.096 |
| **Time from Onse-to-Puncture** | 1 | 1 | 1.001 | 0.552 | 1 | 0.998 | 1 | 0.852 |
| **General Anesthesia** | 1.139 | 0.829 | 1.563 | 0.422 | 1.355 | 0.703 | 2.612 | 0.365 |
| **ASITNSIR<2** | 1.782 | 1.285 | 2.472 | 0.001 | 2.184 | 1.099 | 4.342 | 0.026 |
| **TOAST** |  |  |  |  |  |  |  |  |
| Cardioembolism | 1(Ref) |  |  |  | 1(Ref) |  |  |  |
| Large-artery atherosclerosis | 0.646 | 0.446 | 0.936 | 0.021 | 0.071 | 0.02 | 0.249 | <0.001 |
| other and undermined | 0.709 | 0.382 | 1.315 | 0.275 | 0.03 | 0.004 | 0.23 | 0.001 |
| **Location of Oclussion** |  |  |  |  |  |  |  |  |
| ICA | 1(Ref) |  |  |  | 1(Ref) |  |  |  |
| M1 | 0.596 | 0.413 | 0.861 | 0.006 | 0.964 | 0.431 | 2.156 | 0.929 |
| other | 0.493 | 0.313 | 0.778 | 0.002 | 2.256 | 0.927 | 5.487 | 0.073 |
| **Number of passes** | 1.231 | 1.121 | 1.352 | <0.001 | 1.282 | 0.966 | 1.701 | 0.086 |

ICA: internal carotid artery; M1: first segment of middle cerebral artery; M2: second segment of middle cerebral artery; M3: third segment of middle cerebral artery; A: anterior cerebral artery

The logistic regression analysis was performed to found risk factors of END in each subgroups. Because of the number of patients with occlusion location with A, M1, M2 in group without successful repersion was few, we combined these variables for logistic regression analysis.
